# Adipocyte-specific MC2R overexpression exerts minimal metabolic effects in mice

**DOI:** 10.1186/s42826-026-00296-4

**Published:** 2026-09-04

**Authors:** Sangyi Lim, Edzel Evallo, Dong-il Kim, Min-Jung Park

**Affiliations:** 1 Department of Veterinary Physiology, College of Veterinary Medicine, Chonnam National University, Gwangju, 61186 Korea; 2 College of Veterinary Medicine and BK21 FOUR Program, Chonnam National University, Gwangju, 61186 Korea

**Keywords:** Melanocortin 2 receptor, Obesity, Adeno-associated virus, Metabolism

## Abstract

**Background:**

Obesity is often associated with elevated glucocorticoid (GC) levels mediated by melanocortin 2 receptor (MC2R), the adrenocorticotropic hormone (ACTH) receptor expressed in the adrenal gland. Meanwhile, previous in vitro studies in non-adrenal cells suggest a potential role for MC2R in lipid metabolism in murine primary adipocytes; however, the functional relevance of MC2R in vivo remains poorly understood. Here, we assessed the metabolic effects and therapeutic potential of MC2R in obesity and GC regulation in vivo by generating mice with adipocyte-specific MC2R overexpression using the adeno-associated virus doublef-floxed inverse orientation (AAV-DIO) system.

**Results:**

Our findings show that under basal, high-fat diet (HFD), and stress-induced conditions, MC2R overexpression in adipocytes has minimal effects on thermogenic and lipolytic markers and is insufficient to promote weight reduction in mice. Moreover, adipocyte-specific overexpression of MC2R failed to sequester the ACTH and decrease elevated serum corticosterone levels after exogenous ACTH administration.

**Conclusions:**

These findings demonstrate that adipocyte-specific MC2R overexpression does not induce strong metabolic benefits, suggesting that MC2R-directed gene therapy may not be an effective treatment strategy for obesity.

**Supplementary Information:**

The online version contains supplementary material available at https://doi.org/10.1186/s42826-026-00296-4.

## Background

The worldwide rise in obesity has become a major public health challenge, affecting hundreds of millions of individuals globally. Obesity not only poses significant health risks but also contributes to the development of multiple comorbidities, including cardiovascular disease, diabetes, fatty liver disease, and several types of cancer [1, 2]. Although glucagon-like peptide-1 receptor agonists (GLP-1RAs) have substantially advanced obesity treatment, adverse events such as pancreatitis and potential psychiatric symptoms have been reported [3–5], and emerging evidence raises additional concerns regarding potential metabolic and nutritional consequences of long-term GLP-1RA therapy [6]. Therefore, continued development of alternative anti-obesity therapies with distinct mechanisms of action remains warranted.

The melanocortin system plays a central role in the regulation of energy homeostasis, including satiety and energy expenditure, highlighting its relevance in obesity and related metabolic disorders [7, 8]. This system consists of pro-opiomelanocortin (POMC) neurons, melanocortin receptors (MCRs), and the endogenous antagonist agouti-related peptide (AgRP) [9]. The MCR family comprises five G protein-coupled receptor (GPCR) subtypes (MC1R–MC5R), each exhibiting distinct tissue distributions and ligand specificities. Melanocortin receptors are activated by POMC-derived peptides, including α-, β-, and γ-melanocyte-stimulating hormones (MSHs) as well as adrenocorticotropic hormone (ACTH). Among these ligands, ACTH exclusively activates MC2R, also known as the ACTH receptor [10, 11].

MC2R is predominantly expressed in the adrenal gland, where its activation stimulates the synthesis and secretion of glucocorticoids (GCs)—specifically corticosterone in rodents and cortisol in humans. Excess GC levels have been associated with obesity and metabolic dysfunction, partly through the disruption of lipid homeostasis in adipose tissue [12, 13]. Canonically, ACTH-activated MC2R couples to the G_αs_ subunit, leading to activation of the downstream cyclic adenosine monophosphate/protein kinase A (cAMP/PKA) signaling pathway [14, 15]. Previous in vitro studies using primary murine adipocytes and 3T3-L1 cells have suggested a role for MC2R in lipid metabolism through ACTH-induced lipolysis and thermogenesis mediated by cAMP/PKA signaling [16–21]. However, the functional relevance of MC2R in adipose tissue has not been thoroughly investigated in vivo. These findings suggest that targeting the lipolytic and thermogenic properties of MC2R could offer new approaches to treating lipodystrophy and obesity.

Brown and beige adipocytes drive thermogenesis by metabolizing stored nutrients to generate heat, thereby increasing energy expenditure and reducing lipid accumulation [22, 23]. Accordingly, enhancing thermogenic activity in both brown adipose tissue (BAT) and beige adipocytes within white adipose tissue (WAT) has emerged as a potential therapeutic strategy for obesity. In adipocytes, cAMP/PKA signaling is a key regulator of thermogenic and lipolytic programs. In contrast, elevated GC levels suppress the thermogenic activity of these adipocytes [24–26]. Given that MC2R is the cognate receptor for ACTH and mediates ACTH-induced GC production, adipocyte-specific expression of MC2R may influence circulating ACTH availability and modulate downstream cAMP/PKA signaling in adipose tissue. Therefore, we hypothesized that adipocyte-specific MC2R expression could modulate GC-associated metabolic responses while enhancing thermogenic and lipolytic activity through cAMP/PKA signaling. To investigate this possibility in vivo, we generated mice with adipocyte-specific MC2R overexpression using the adeno-associated virus double-floxed inverse orientation (AAV-DIO) system.

## Methods

### Mice

All animal experiments conducted in this study were approved by the Institutional Animal Care and Use Committee of Chonnam National University (#CNU IACUC-YB-2025-62). Mice were housed at 22 °C with 50 ± 5% relative humidity in a 12-hour light/dark cycle with ad libitum access to food and water. To generate AMOE mice, adiponectin-Cre recombinase (AQ-Cre, Jackson Lab, #028020) and wild-type mice were intraperitoneally injected with the AAV-DIO-MC2R virus (500 µL of 5 × 10^12^ virus particles per mouse). For the diet-induced obesity models, mice were fed a HFD with 60% of the calories from fat (#D12492, Research Diets). For the cold exposure experiments, five weeks after AAV-DIO-MC2R injection, mice were singly housed at 4 °C for seven days, and the cold stress response was assessed. For the exogenous administration of ACTH, ACTH fragment 1–24 (#A0298, Sigma-Aldrich) was injected intraperitoneally at 09:00 AM, considering the circadian pattern of circulating corticosterone levels in mice [27], at a dose of 57 ng per mouse per injection, following a previously described method [28]. On day 20, mice were administered ACTH at 09:00 AM, fasted for six hours, and sacrificed in the afternoon on the same day.

### Virus preparation

We used an AAV-DIO system to overexpress MC2R specifically in adipocytes. The plasmid construct AAV-FLEX-mMC2R-FLAG was designed using Vectorbuilder (IL, USA) and was co-transfected with pAAV2/8 (#112864, Addgene) and helper plasmid pAdDelta6 (#112867, Addgene) into HEK-293T cells using polyethylenimine in a 1:1:1 molar ratio. The cells were harvested and resuspended in AAV lysis buffer (150 mM NaCl and 20 mM Tris, pH 8.0) 60 h after transfection. Virus particles were extracted through lysis in three cycles of freezing and thawing, followed by incubation with Benzonase (#8263, Sigma-Aldrich). The virus was then purified using an iodixanol gradient solution and a high-speed centrifuge. Shortly after, the concentration of the viral fraction was performed using the Amicon Ultra 15 Centrifugal Filter Unit (#UFC910096, Sigma-Aldrich). This was followed by two washes with phosphate-buffered saline (PBS) containing 0.1% Poloxamer 188 (#P5556, Sigma-Aldrich) and three washes with PBS alone. The virus was added to 5 µL aliquots and initially incubated with DNase, followed by proteinase K for further purification. The virus concentration was determined using quantitative real-time PCR (#CFX96, Bio-Rad).

### mRNA quantification

Frozen mouse adipose tissue samples were lysed in a Tri-solution (#TS200-001, Biosolution). To extract total RNA from mice fed a HFD, the inguinal WAT (iWAT) and epididymal WAT (eWAT) were processed using Tri-solution and the RNeasy kit (#74106, Qiagen). The sample homogenates were centrifuged at 4 °C and 13,000 rpm for 10 min. The RNA-rich layer was collected and mixed with chloroform in a 1:5 ratio, then centrifuged at 4 °C and 13,000 rpm for 10 min. The uppermost layer of the resulting mixture was collected, combined with an equal volume of 70% ethanol, and transferred to a RNeasy mini spin column. Next, RNA extraction was performed according to the manufacturer’s instructions. cDNA was synthesized from 2.5 µg of total RNA using Moloney murine leukemia virus reverse transcriptase (#28025013, Thermo Fisher Scientific). Quantitative real-time PCR was performed using the CFX96 system (Bio-Rad), and relative gene expression levels were normalized to *TATA-box binding protein (Tbp)* mRNA. Primer sequences used in this study are provided in Supplementary Table 1.

### Immunoblot analysis

Frozen adipose tissue samples were homogenized using radioimmunoprecipitation assay (RIPA) buffer (140 mM NaCl, 10 mM Tris at pH 8.0, 0.1% sodium deoxycholate, 0.1% sodium dodecyl sulfate, and 1% Triton X-100) containing a protease inhibitor (#ab201112, Abcam). The protein concentration for each sample was quantified using DC protein assay reagents (#5000116, Bio-Rad). Standardized protein samples were then subjected to acetone precipitation as previously reported [29], and the pellets were resuspended in sodium dodecyl sulfate (SDS) sample buffer (60 mM Tris, pH 6.8, 25% glycerol, 0.1% bromophenol blue, 2% SDS, 2.5% 2-mercaptoethanol). SDS-polyacrylamide gel electrophoresis (PAGE) was performed to resolve the solubilized proteins, which were electrophoretically transferred onto nitrocellulose membranes. The blots were incubated in the indicated primary antibodies against FLAG (#PA1-984B, ThermoFisher Scientific), HSP90 (#4874, Cell Signaling Technology), p-PKA substrates (#9621, Cell Signaling Technology), p-PLIN1 (#4856, Vala Sciences), p-HSL (#4126, Cell Signaling Technology), UCP1 (#ab10983, Abcam), β-actin (#4967, Cell Signaling Technology), and OXPHOS (#ab110413; Abcam), according to the manufacturer’s instructions, at 4 ℃ with constant rocking overnight. Protein bands were visualized using a luminescent image analyzer (ImageQuant LAS 4000 mini; GE Healthcare, IL, USA), and band intensity was quantified using ImageJ.

### Histological analysis

Shortly after euthanasia, adipose tissues were collected and fixed in 10% neutral buffered formalin (NBF). Paraffin-embedded tissue blocks were then cut into 5 mm (BAT) or 7 mm (iWAT and eWAT) thick sections and stained with hematoxylin and eosin.

### ELISA assay

Corticosterone levels were measured in mouse serum using the corticosterone ELISA kit (#CSB-E07969m 96T) according to the manufacturer’s instructions. Briefly, serum samples were diluted and added to pre-coated wells in the ELISA plate. Following incubation, the absorbance of the samples was measured at 450 nm, and the concentration was then quantified using a standard curve.

### Statistical analysis

All data collected from independent biological samples in this study are reported as the mean ± standard error of the mean (SEM). Statistical significance between two groups was determined using Student’s t-test. For analyses involving multiple factors, the statistical tests used are indicated in the corresponding figure legends. GraphPad Prism (version 9.3.1, GraphPad Software, CA, USA) was used for statistical analysis and data plotting.

## Results

### Adipocyte-specific MC2R overexpression exerts minimal metabolic effects

**Fig. 1:**
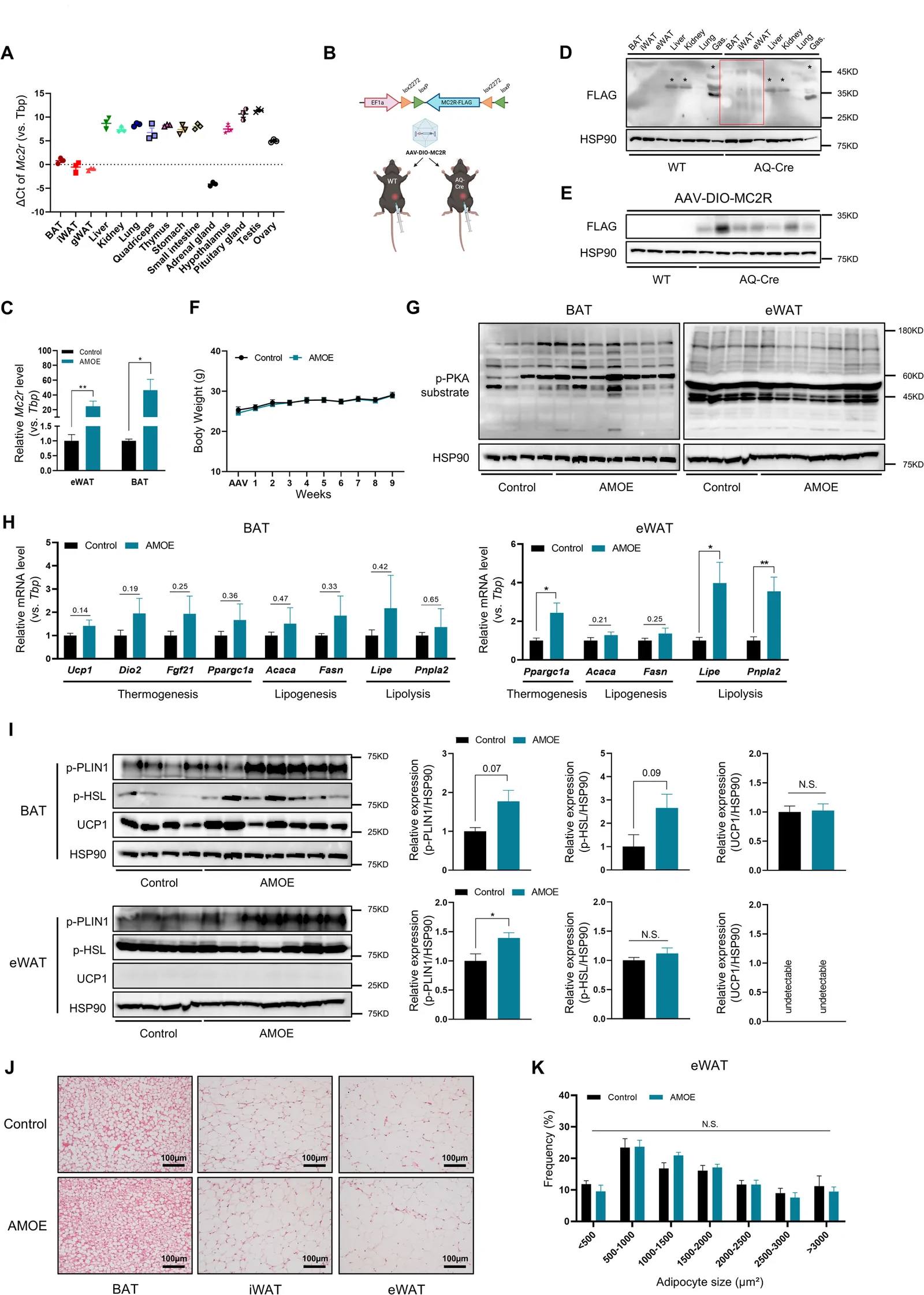
Adipocyte-specific MC2R overexpression exerts minimal metabolic effects. (**A**) ΔCt values of the Mc2r gene compared to Tbp are shown as relative Mc2r expression in different tissues of mice (n = 3). (**B**) Schematic diagram of MC2R overexpression in mouse adipocytes. Mice were intraperitoneally injected with AAV-DIO-MC2R. (**C**) mRNA expression levels of Mc2r in eWAT and BAT from age- and sex-matched control and AMOE mice under chow diet (CD) conditions (Control, n = 6; AMOE, n = 7). C57BL/6J littermate mice were used as controls. (**D**) Immunoblot confirmation of adipose tissue-specific overexpression of MC2R between wild-type (WT) and AQ-Cre mice. FLAG detection is presented in a red box. *Asterisks indicate non-specific bands. Gas = gastrocnemius. (**E**) Immunoblot of the MC2R overexpression in eWAT of control (n = 4) and AMOE (n = 7) mice fed a normal chow diet 10 weeks post-injection. HSP90 was used as a loading control. (**F**) Body weight representation of AMOE and control mice under normal chow diet (control, n = 9; AMOE, n = 7). (**G**) Immunoblot of the phosphorylation levels of PKA substrates in mice (control, n = 4; AMOE, n = 7). HSP90 was used as a loading control. (**H**) mRNA expression levels of the indicated genes in the BAT and eWAT of mice (control, n = 9; AMOE, n = 7). (**I**) Immunoblot and densitometry analysis of the indicated proteins in BAT and eWAT of control and AMOE mice (control, n = 4; AMOE, n = 7). (**J**) Representative histological images of BAT, iWAT, and eWAT in control and AMOE mice fed a normal chow diet (control, n = 9; AMOE, n = 7), scale bar = 100 μm. K) Adipocyte size distribution in eWAT from control and AMOE mice. A student’s t-test (**C**, **H**, **I**) and two-way ANOVA (**F**, **K**) were used to conduct statistical analysis, with the values presented as the mean ± SEM. Differences between groups are denoted as * p < 0.05, ** p < 0.01, N.S. = not significant

Consistent with previous reports [20, 30], the mRNA expression of *Mc2r* was more prominent in the adrenal gland and adipose tissues compared to other tissues (Fig. 1A). To investigate whether adipocyte-specific expression of MC2R could ultimately exert anti-obesity effects in vivo, we selectively overexpressed FLAG-tagged MC2R in mouse adipocytes using a Cre-dependent AAV-DIO system driven by the adiponectin (AQ) promoter (Fig. 1B) [31]. As expected, *Mc2r* mRNA levels were markedly increased, reaching approximately 20-fold in epididymal WAT (eWAT) and 40-fold in BAT (Fig. 1C). We next validated the tissue specificity of the system by examining FLAG expression across multiple tissues to assess potential off-target expression. Consistent with previous studies using adiponectin-Cre-driven AAV-DIO systems [32], Western blot analysis revealed FLAG expression in the lysates from three adipose tissues of the AQ-Cre mice, whereas FLAG expression was undetectable in the wild-type (WT) littermates (Fig. 1D). Adipocyte-specific overexpression of MC2R was further confirmed in the eWAT of Cre mice (hereafter referred to as AMOE) but not in WT littermates (Fig. 1E). MC2R is known to require melanocortin receptor accessory protein (MRAP) for proper trafficking and functional activation [33, 34]. Analysis of publicly available databases revealed that *Mrap* is highly and specifically expressed in adipocytes within adipose tissues (#GSE176171), suggesting that adipocytes may possess the accessory machinery required to support MC2R signaling (Figure S1). Nevertheless, under a standard chow diet, no significant difference in body weight was observed between the two groups (Fig. 1F). To further evaluate whether adipocyte-specific MC2R overexpression activates cAMP/PKA signaling, we assessed the phosphorylation of PKA substrates in adipose tissues, but no significant differences were observed between control and AMOE mice (Fig. 1G). In BAT, although no significant changes were observed in the expression of thermogenic, lipogenic, or lipolytic genes (Fig. 1H), increased trends in the phosphorylation of perilipin1 (PLIN1) and hormone-sensitive lipase (HSL) (Fig. 1I), along with a marked reduction in lipid accumulation (Fig. 1J), were observed in AMOE mice. In contrast, eWAT exhibited increased expression of *Ppargc1a* and lipolysis-related genes (Fig. 1H), along with significantly elevated PLIN1 phosphorylation (Fig. 1I); however, these changes were not accompanied by alterations in adipocyte size (Fig. 1J and K). These findings suggest that adipocyte-specific MC2R overexpression induces subtle downstream activation of lipolysis, particularly in BAT in vivo, although these alterations were insufficient to reduce body weight.

### Adipocyte-specific MC2R overexpression does not protect mice from diet-induced obesity

**Fig. 2:**
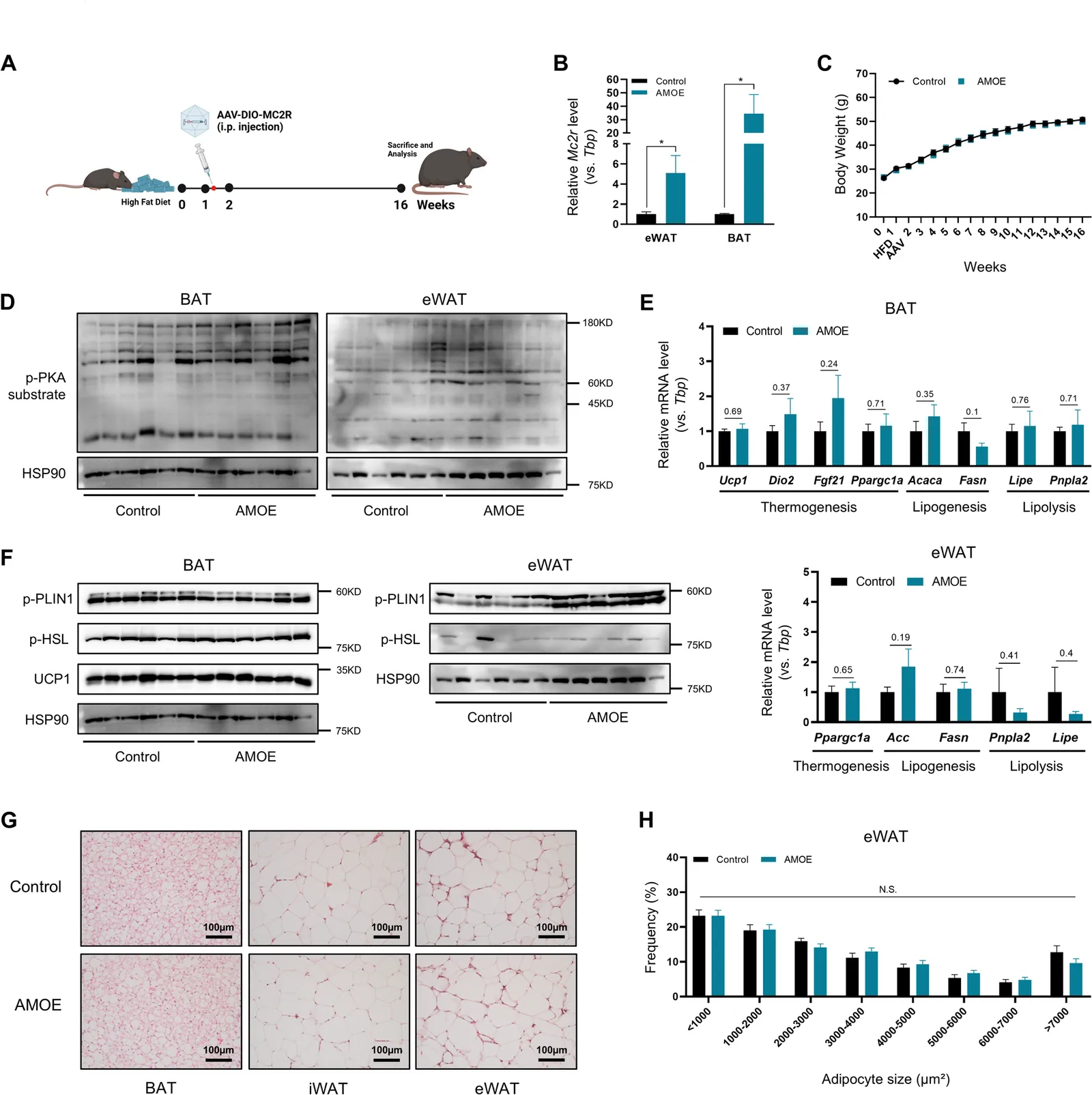
Adipocyte-specific MC2R overexpression does not protect mice from diet-induced obesity. (**A**) Schematic representation of the HFD experiment between control and AMOE mice. (**B**) mRNA expression levels of Mc2r in eWAT and BAT from age- and sex-matched control and AMOE mice under HFD conditions (Control, n = 6; AMOE, n = 6). C57BL/6J littermate mice were used as controls. (**C**) Body weights of mice fed a HFD (control, n = 11; AMOE, n = 10). Mice were intraperitoneally injected with AAV-DIO-MC2R after 1 week of a HFD over 16 weeks of feeding. (**D**) Immunoblot showing phosphorylation levels of PKA substrates of mouse groups fed a HFD (control, n = 6; AMOE, n = 6). HSP90 was used as a loading control. (**E**) mRNA expression levels of the indicated genes in the BAT and eWAT of the mice fed a HFD (control, n = 5; AMOE, n = 6). (**F**) Immunoblot analysis of the indicated proteins in BAT and eWAT of control and AMOE mice fed a HFD (control, n = 6; AMOE, n = 6). HSP90 was used as a loading control. (**G**) Representative histological images of BAT, iWAT, and eWAT in control and AMOE mice fed a HFD (control, n = 5; AMOE, n = 5), scale bar = 100 μm. (**H**) Adipocyte size distribution in eWAT from control and AMOE mice. A student’s t-test (**B**, **E**) and two-way ANOVA (**C**, **H**) were used to conduct statistical analysis, with values presented as the mean ± SEM. Differences between groups are denoted as * p < 0.05, N.S. = not significant

Chronic overnutrition caused by excessive caloric intake from a high-fat diet (HFD) disrupts lipid metabolism and contributes to the development of obesity [35]. To evaluate the impact of adipocyte-specific MC2R overexpression on diet-induced obesity, mice were fed a HFD for 16 weeks (Fig. 2A). Despite approximately 5-fold and 30-fold increases in *Mc2r* mRNA expression in eWAT and BAT, respectively, both control and AMOE mice exhibited comparable increases in body weight during HFD feeding (Fig. 2B and C). In addition, neither PKA activity nor the expression of thermogenic, lipolytic, and lipogenic genes in BAT and eWAT was significantly altered in AMOE mice (Fig. 2D and E). Nevertheless, increased PLIN1 phosphorylation was observed in the eWAT, but not in the BAT, of AMOE mice (Fig. 2F). However, histological analysis of eWAT revealed no significant differences in adipocyte morphology or size between the two groups (Fig. 2G and H). Collectively, these findings suggest that adipocyte-specific MC2R overexpression is insufficient to protect mice from diet-induced obesity.

### Adipocyte-specific MC2R overexpression does not enhance metabolic responses to stress

**Fig. 3:**
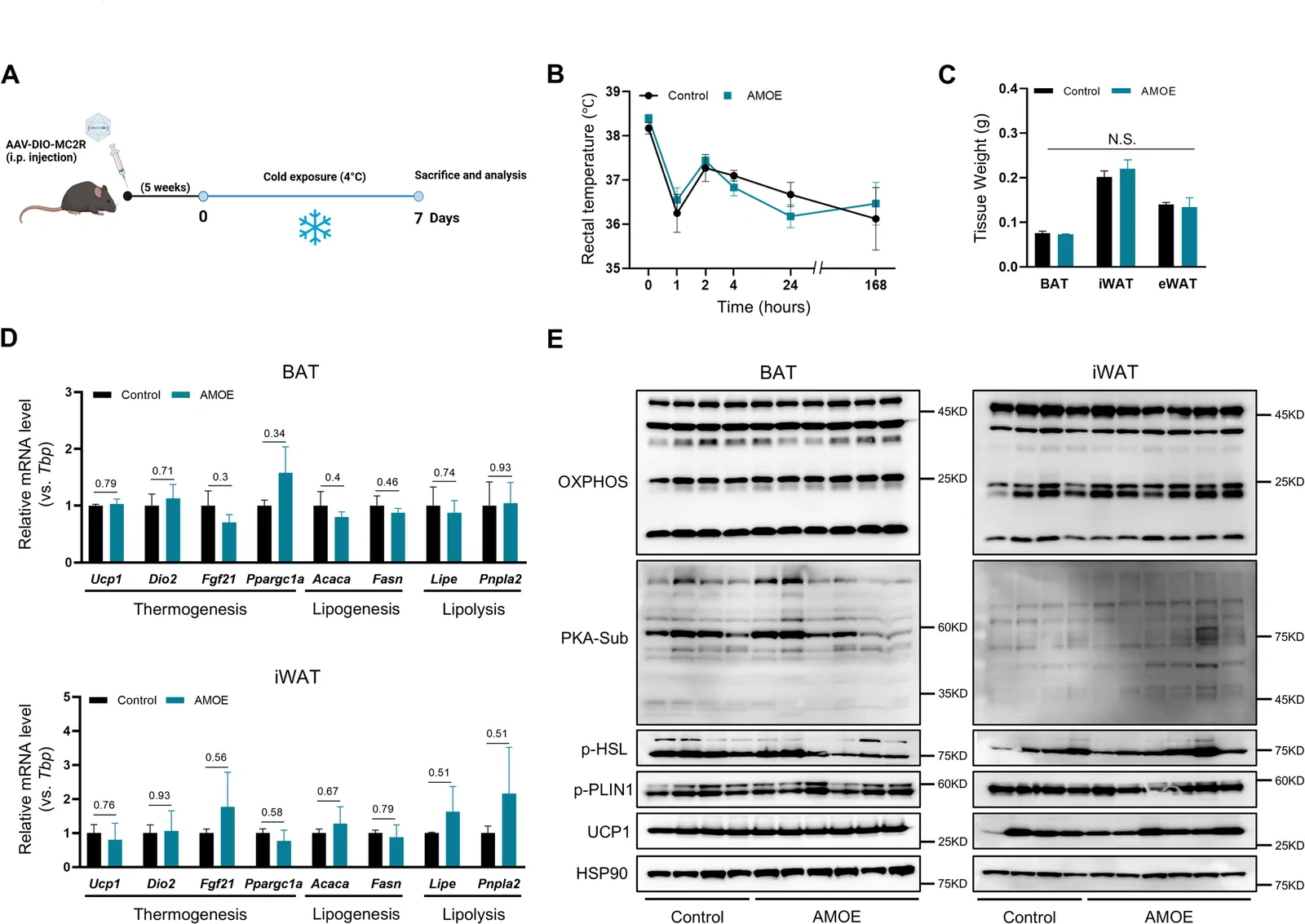
Adipocyte-specific MC2R overexpression does not enhance thermogenic responses to cold exposure. (**A**) Schematic diagram of the cold exposure experiment. (**B**) Rectal temperatures of mice obtained using a rectal thermometer (control, n = 4; AMOE, n = 6). (**C**) Adipose tissue weights in mice after exposure to 4 °C for 7 days (control, n = 4; AMOE, n = 6). (**D**) mRNA expression levels of the indicated genes in the BAT and iWAT of mice after exposure to 4 °C for 7 days (control, n = 4; AMOE, n = 6). (**E**) Immunoblot analysis of the indicated proteins in BAT and iWAT after exposure to 4 °C for 7 days (control, n = 4; AMOE, n = 6). HSP90 was used as a loading control. A student’s t-test (**C**, **D**) and two-way ANOVA (**B**) were used to conduct statistical analysis, with values presented as the mean ± SEM. N.S. = not significant

We hypothesized that MC2R may regulate thermogenesis in response to cold stress-induced ACTH release [36, 37]. To assess whether adipocyte-specific MC2R overexpression enhances thermogenic responses, mice were exposed to cold conditions (4 °C) for seven days (Fig. 3A). However, no significant differences in rectal temperature or adipose tissue weight were observed between control and AMOE littermates following cold exposure (Fig. 3B and C). Furthermore, the expression of thermogenic, lipolytic, and lipogenic genes remained largely unchanged in both BAT and inguinal WAT (iWAT) of AMOE mice (Fig. 3D). Similarly, no significant changes were observed in the protein levels of oxidative phosphorylation complexes (OXPHOS) or uncoupling protein 1 (UCP1), a key thermogenic protein, or in the phosphorylation levels of HSL and PLIN1. (Fig. 3E).

**Fig. 4:**
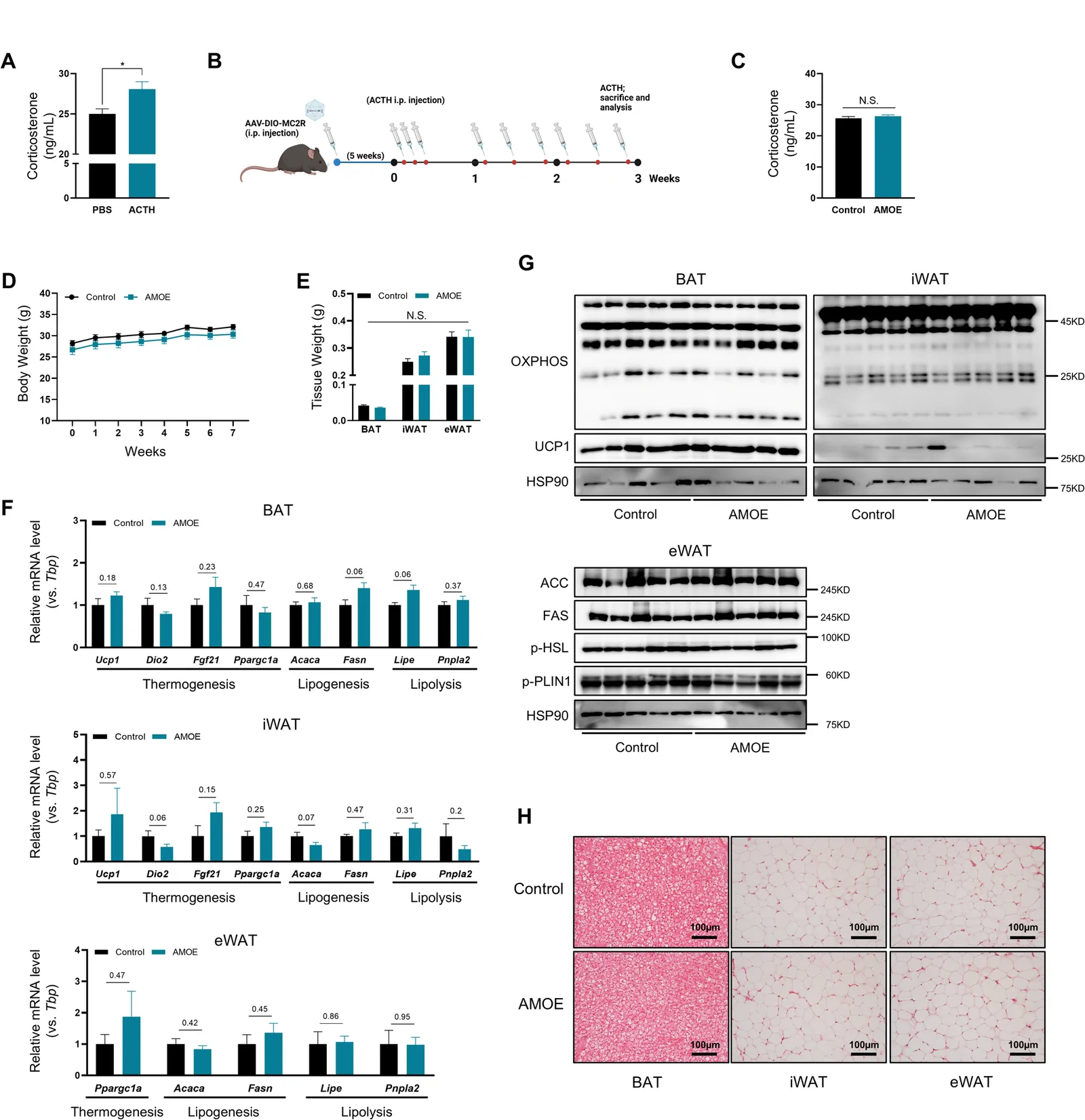
Adipocyte-specific MC2R overexpression does not affect metabolic responses to ACTH administration. (**A**) Serum corticosterone levels of control male mice injected with PBS (vehicle, n = 4) or ATCH (n = 4) according to the same injection scheme shown in (**B**). (**B**) Schematic diagram of ACTH administration in AMOE male mice. At 5 weeks post-AAV-DIO-MC2R injection, ACTH was administered intraperitoneally daily for three days. In the subsequent weeks, three injections were administered weekly until day 20. (**C**) Serum corticosterone levels in control and AMOE male mice after ACTH injection (control, n = 5; AMOE, n = 5). (**D**) Body weights of AMOE male mice after ACTH injection (control, n = 5; AMOE, n = 10). (**E**) Adipose tissue weights of AMOE mice after ACTH injection (control, n = 5; AMOE, n = 10). (**F**) mRNA expression levels of the indicated genes in the adipose tissues of AMOE male mice after ACTH injection (control, n = 5; AMOE, n = 10). (**G**) Immunoblot analysis of the indicated proteins in BAT, iWAT, and eWAT of AMOE male mice after ACTH injection (control, n = 5; AMOE, n = 5). HSP90 was used as a loading control. (**H**) Representative histological images of BAT, iWAT, and eWAT of AMOE male mice after ACTH injection (control, n = 5; AMOE, n = 5). Scale bar = 100 μm. A student’s t-test (**A**, **C**, **E**, **F**) and two-way ANOVA (**D**) were used to conduct statistical analysis, with values presented as the mean ± SEM. Differences between groups are denoted as * p < 0.05, N.S. = not significant

Next, we evaluated the physiological response to exogenous ACTH administration in mice. As expected, ACTH injection increased serum corticosterone levels compared with vehicle treatment (Fig. 4A). Because MC2R is the cognate receptor for ACTH, its overexpression in adipocytes may create an additional ACTH-binding compartment outside the adrenal gland. We therefore hypothesized that MC2R-overexpressing adipose tissues could function as a peripheral ACTH sink, potentially limiting the adrenal response to circulating ACTH. To test this hypothesis, mice received intraperitoneal ACTH injections daily for the first three days, followed by injections three times weekly until day 20 (Fig. 4B) [28]. However, no significant differences in circulating corticosterone levels were observed between control and AMOE mice following ACTH administration (Fig. 4C). In addition, body weight and adipose tissue weight were comparable between the two groups (Fig. 4D and E). Furthermore, no significant differences were observed in the mRNA and protein expression levels of lipogenic, lipolytic, and thermogenic markers in adipose tissues between control and AMOE mice (Fig. 4F and G). Consistently, histological analysis revealed similar adipocyte morphology and size in both groups (Fig. 4H). Together, these findings suggest that adipocyte-specific MC2R overexpression is insufficient to attenuate the ACTH-induced corticosterone response or promote PKA-mediated lipolysis and thermogenesis in adipose tissues.

Given the known sex-dependent differences in adipose tissue metabolism and stress responses in mice [38], we additionally assessed the metabolic effects of exogenous ACTH administration in female mice. Similar to the findings in male mice, no significant differences were observed in body weight, adipose tissue weight, or the mRNA and protein expression levels in BAT, iWAT, and eWAT between AMOE and control mice (Figure S2). These findings suggest that adipocyte-specific MC2R overexpression has minimal metabolic effects in either sex under exogenous ACTH stimulation.

## Discussion

Obesity is a complex metabolic disorder influenced by sustained energy imbalance, altered endocrine signaling, and impaired adipose tissue function. As adipose tissues play central roles in lipid storage, lipid mobilization, thermogenesis, and systemic energy homeostasis, therapeutic strategies targeting adipose tissue metabolism have gained considerable attention. In parallel, AAV-mediated gene therapy has emerged as an attractive experimental and therapeutic platform because of the low immunogenicity, high cell transduction efficiency, and long-term gene expression capacity of AAV vectors [39]. As our understanding of the molecular mechanisms regulating adipose tissue metabolism continues to expand, AAV-mediated gene delivery offers a useful strategy to investigate and potentially modulate genes involved in lipid metabolism and thermogenesis [39, 40].

The melanocortin system is a key regulator of energy metabolism, and melanocortin receptors have been implicated in both central and peripheral metabolic regulation [7, 8, 16, 19]. Among these receptors, MC2R is unique because ACTH is its cognate ligand. In the adrenal cortex, ACTH activates MC2R to induce GC synthesis through canonical G_αs_-cAMP/PKA signaling [14, 15, 30]. In adipocytes, previous studies have suggested that ACTH can also stimulate lipolysis through MC2R-dependent activation of cAMP/PKA signaling [16–20]. More recently, Schnabl et al. demonstrated that ACTH can act as a non-adrenergic activator of murine brown adipocytes, promoting lipolysis and UCP1-mediated respiration through MC2R-dependent signaling [41]. Importantly, the same study showed that GCs exert opposing effects on UCP1-mediated respiration in brown adipocytes, highlighting a complex relationship among ACTH, GCs, and adipocyte thermogenic function [41]. These findings provided an important context for our study, in which we tested whether adipocyte-specific MC2R overexpression could enhance ACTH-responsive lipolytic and thermogenic signaling while potentially attenuating ACTH-induced GC elevation.

In the present study, AAV-mediated adipocyte-specific MC2R overexpression induced only modest molecular changes under basal conditions. Although AMOE mice showed increased PLIN1 phosphorylation and reduced lipid accumulation in BAT, these changes were not accompanied by broad activation of PKA substrates, robust induction of thermogenic genes, increased UCP1 protein expression, or reduced body weight. Similarly, eWAT showed increased expression of selected lipolysis-related genes and elevated PLIN1 phosphorylation, but these molecular changes did not result in altered adipocyte size or whole-body adiposity. These findings suggest that adipocyte-specific MC2R overexpression can induce limited downstream activation of lipolytic signaling in vivo, but this effect is insufficient to drive a coordinated thermogenic or anti-obesity phenotype under basal conditions.

The limited metabolic effect of MC2R overexpression became more evident under HFD-induced obesity. Despite increased *Mc2r* expression in adipose tissues, AMOE mice were not protected from diet-induced body weight gain or adipose tissue expansion. Moreover, HFD-fed AMOE mice did not exhibit significant increases in thermogenic or lipolytic gene expression, global PKA activity, UCP1 expression, or adipose tissue remodeling. Although PLIN1 phosphorylation was increased in eWAT, this isolated molecular change did not result in measurable metabolic benefit. These results suggest that, under chronic obesogenic conditions, AAV-mediated adipocyte-specific MC2R overexpression is not sufficient to induce meaningful adipose tissue remodeling or reduce body weight.

We further examined whether adipocyte-specific MC2R overexpression could enhance metabolic responses under physiologically demanding conditions. Cold exposure is a strong activator of brown fat thermogenesis, and previous work has suggested that ACTH may contribute to non-adrenergic regulation of brown adipocyte metabolism [19, 36, 37, 41]. However, in our cold exposure experiment, AMOE mice did not show improved cold tolerance, reduced adiposity, increased OXPHOS complex abundance, or enhanced UCP1 and lipolytic protein expression in BAT or iWAT. These findings indicate that adipocyte-specific MC2R overexpression does not substantially augment cold-induced thermogenic responses in vivo. One possible explanation is that cold-induced thermogenesis is primarily driven by sympathetic β-adrenergic signaling, and this pathway may already sufficiently engage cAMP/PKA-dependent thermogenic machinery. In such a context, additional MC2R expression may provide limited additive benefit.

We also tested whether MC2R-overexpressing adipose tissue could function as a peripheral ACTH-buffering compartment. Because MC2R is the cognate receptor for ACTH, we hypothesized that MC2R overexpression in adipocytes might reduce ACTH availability to the adrenal gland and thereby attenuate corticosterone production. However, serum corticosterone levels after ACTH administration were comparable between control and AMOE mice. In addition, body weight, adipose tissue weight, adipocyte morphology, and thermogenic/lipolytic markers were largely unchanged. These findings suggest that adipocyte-specific MC2R overexpression does not meaningfully attenuate ACTH-induced corticosterone production under the conditions tested in this study. This may reflect the high sensitivity and specialized steroidogenic capacity of adrenal cortical cells, which may dominate the systemic corticosterone response to ACTH.

One limitation of the ACTH administration experiment is that the injected ACTH dose may have produced a strong adrenal stimulus. In this study, ACTH was administered as repeated injections, which may not fully recapitulate physiological patterns of ACTH secretion. Therefore, if the administered ACTH dose was sufficient to produce near-maximal adrenal MC2R activation, subtle differences in ACTH availability caused by adipocyte MC2R overexpression could have been masked. Future studies using lower ACTH doses or dose-response approaches may help determine whether adipocyte MC2R overexpression can influence corticosterone production under conditions of submaximal adrenal stimulation [42].

## Conclusions

In summary, this study demonstrates that AAV-mediated adipocyte-specific MC2R overexpression produces only modest molecular changes in adipose tissues and does not confer clear metabolic benefits under basal, obesogenic, cold stress, or ACTH-stimulated conditions. These findings do not exclude a physiological role for ACTH-MC2R signaling in adipocytes, particularly in light of previous evidence showing ACTH-dependent activation of brown adipocyte metabolism. Rather, our results indicate that increasing MC2R expression in adipocytes through an AAV-mediated gene delivery strategy is insufficient to robustly enhance adipose thermogenesis, prevent diet-induced obesity, or attenuate ACTH-induced corticosterone production. Thus, adipocyte-directed MC2R overexpression appears to have limited therapeutic potential as a standalone gene therapy approach for obesity or GC modulation.

## Supplementary Information

Below is the link to the electronic supplementary material.

Supplementary Material 1: Supplementary Table 1 summarizes the primer sequences used for mRNA quantification. Supplementary Figure S1. Single-cell analysis of Mrap expression in mouse WAT. UMAP visualization of Mrap expression in mouse WAT using publicly available single-cell RNA-seq data from “A single cell atlas of human and mouse white adipose tissue” (Single Cell Portal dataset SCP1376; GSE176171). Supplementary Figure S2. Adipocyte-specific MC2R overexpression in female mice does not induce metabolic changes following exogenous ACTH administration. A) Body weight measurements of AMOE female mice after exogenous ACTH administration (control, n = 7; AMOE, n = 11). B) Adipose tissue weights of AMOE female mice injected with exogenous ACTH (control, n = 7; AMOE, n = 11). C) mRNA expression levels of the indicated genes in the adipose tissues of AMOE female mice after exogenous ACTH administration (control, n = 7; AMOE, n = 7). D) Immunoblot analysis of the indicated proteins in the eWAT of AMOE female mice after ACTH injection (control, n = 5; AMOE, n = 5). β-actin was used as a loading control. A student’s t-test (B, C) and two-way ANOVA (A) were used to conduct statistical analysis, with values presented as the mean ± SEM. Differences between groups are denoted as * p < 0.05, N.S. = not significant

Supplementary Material 2

## Data Availability

No datasets were generated or analysed during the current study.
