# Supplementary material for: Adipocyte-specific MC2R overexpression exerts minimal metabolic effects in mice
Source: Lab Anim Res. 2026 Sep 4;42:34. doi: 10.1186/s42826-026-00296-4 (PMC13543462; doi:10.1186/s42826-026-00296-4)

Figure 1D

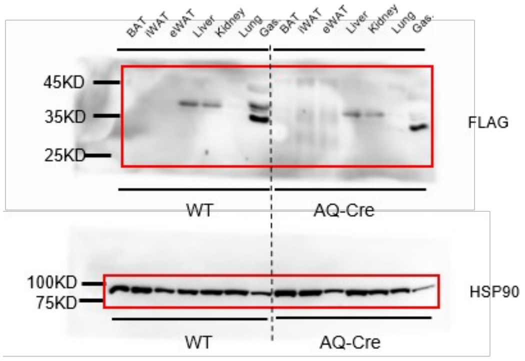

Figure 1E

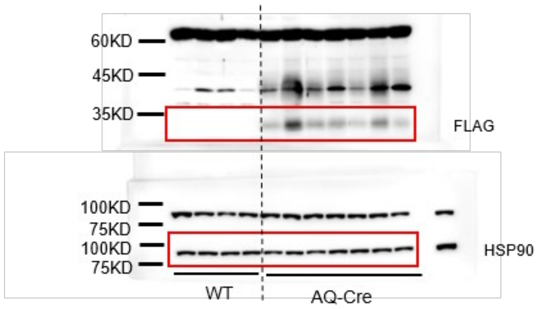

Figure 1G

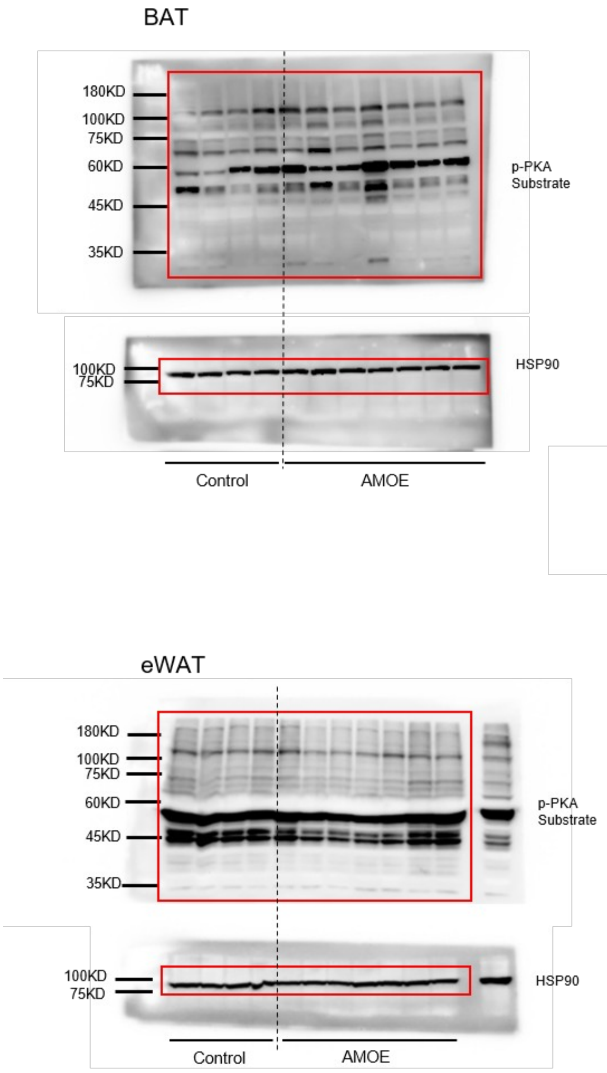

Figure 1I

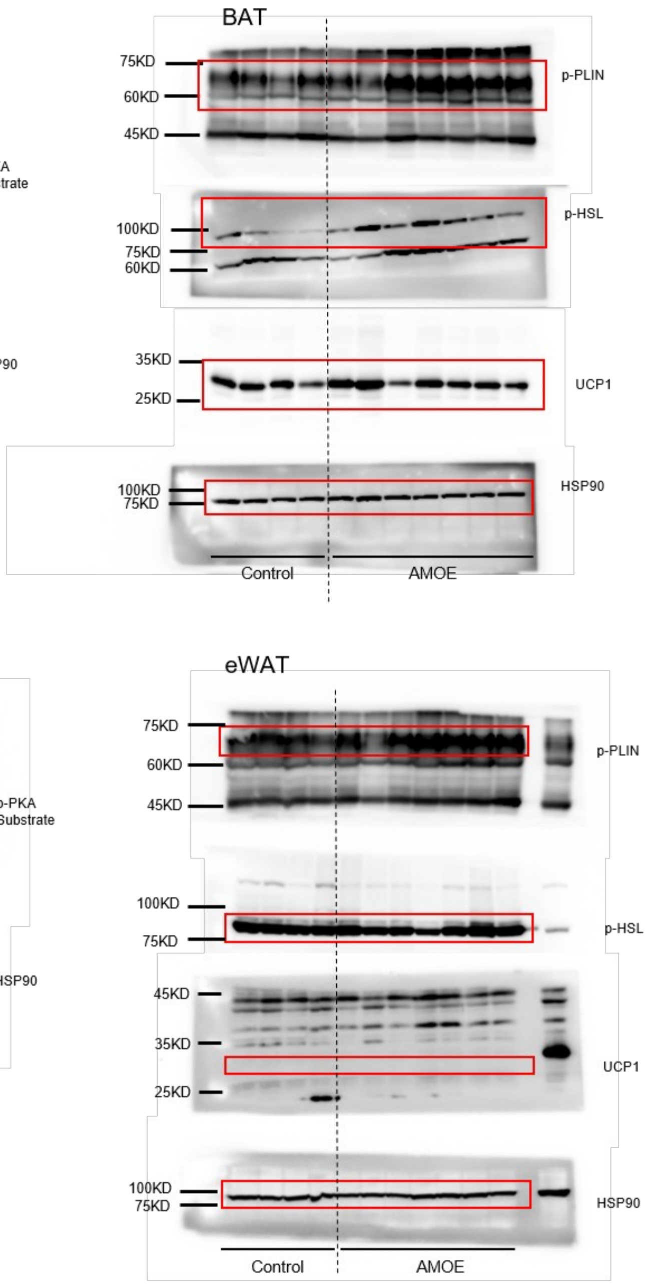

Figure 2D

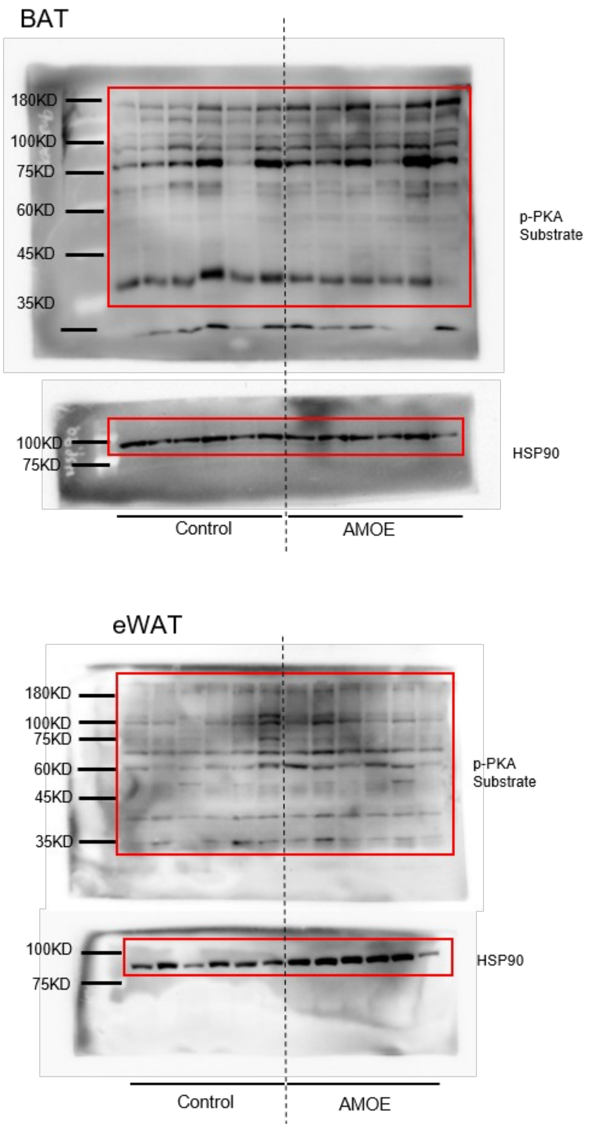

Figure 2F

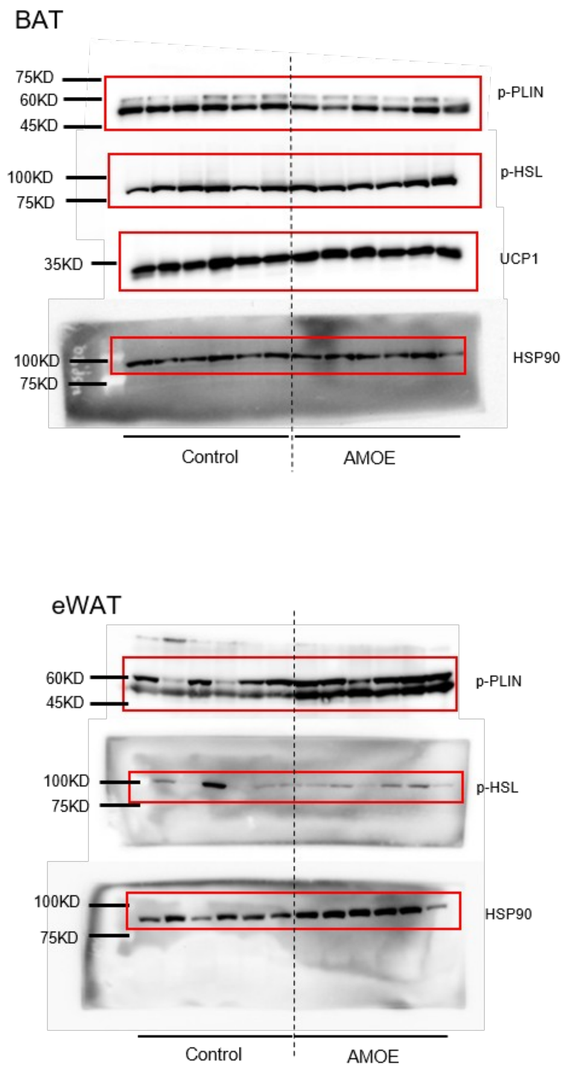

Figure 3E

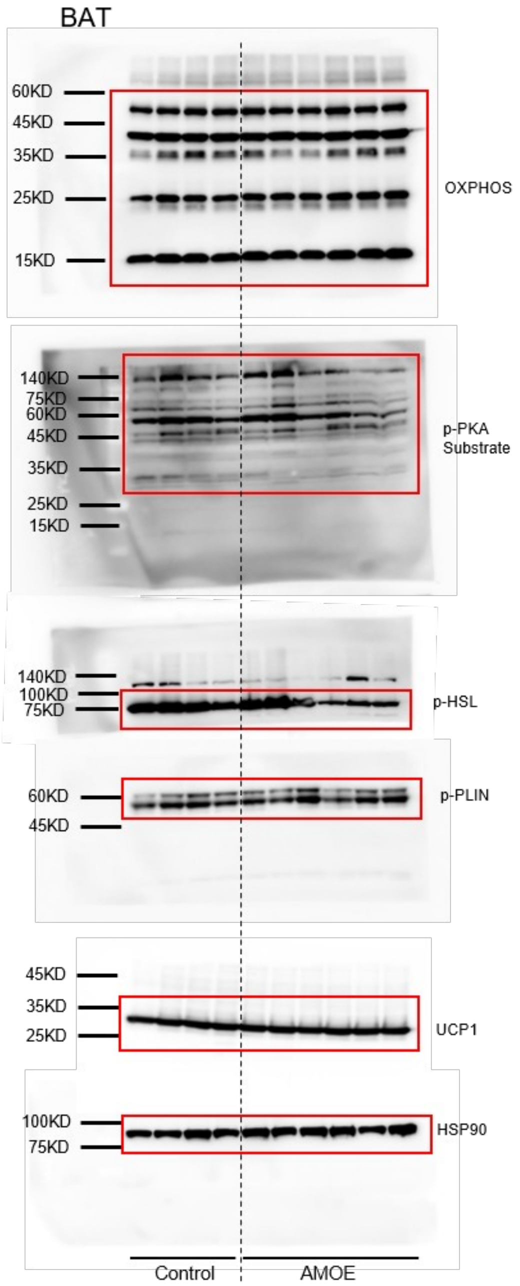

BAT
60KD
45KD
35KD
25KD
15KD
OXPHOS
140KD
75KD
60KD
45KD
35KD
25KD
15KD
p-PKA
Substrate
140KD
100KD
75KD
p-HSL
60KD
45KD
p-PLIN
45KD
35KD
25KD
UCP1
100KD
75KD
HSP90
Control
AMOE

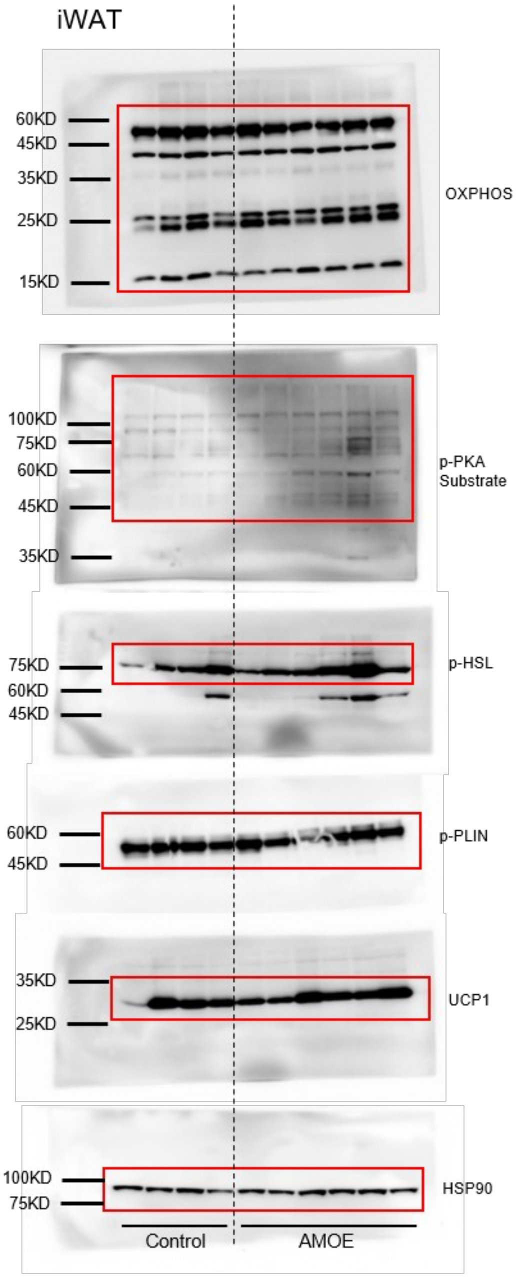

iWAT
60KD
45KD
35KD
25KD
15KD
OXPHOS
100KD
75KD
60KD
45KD
35KD
p-PKA
Substrate
75KD
60KD
45KD
p-HSL
60KD
45KD
p-PLIN
35KD
25KD
UCP1
100KD
75KD
HSP90
Control
AMOE

Figure 4G

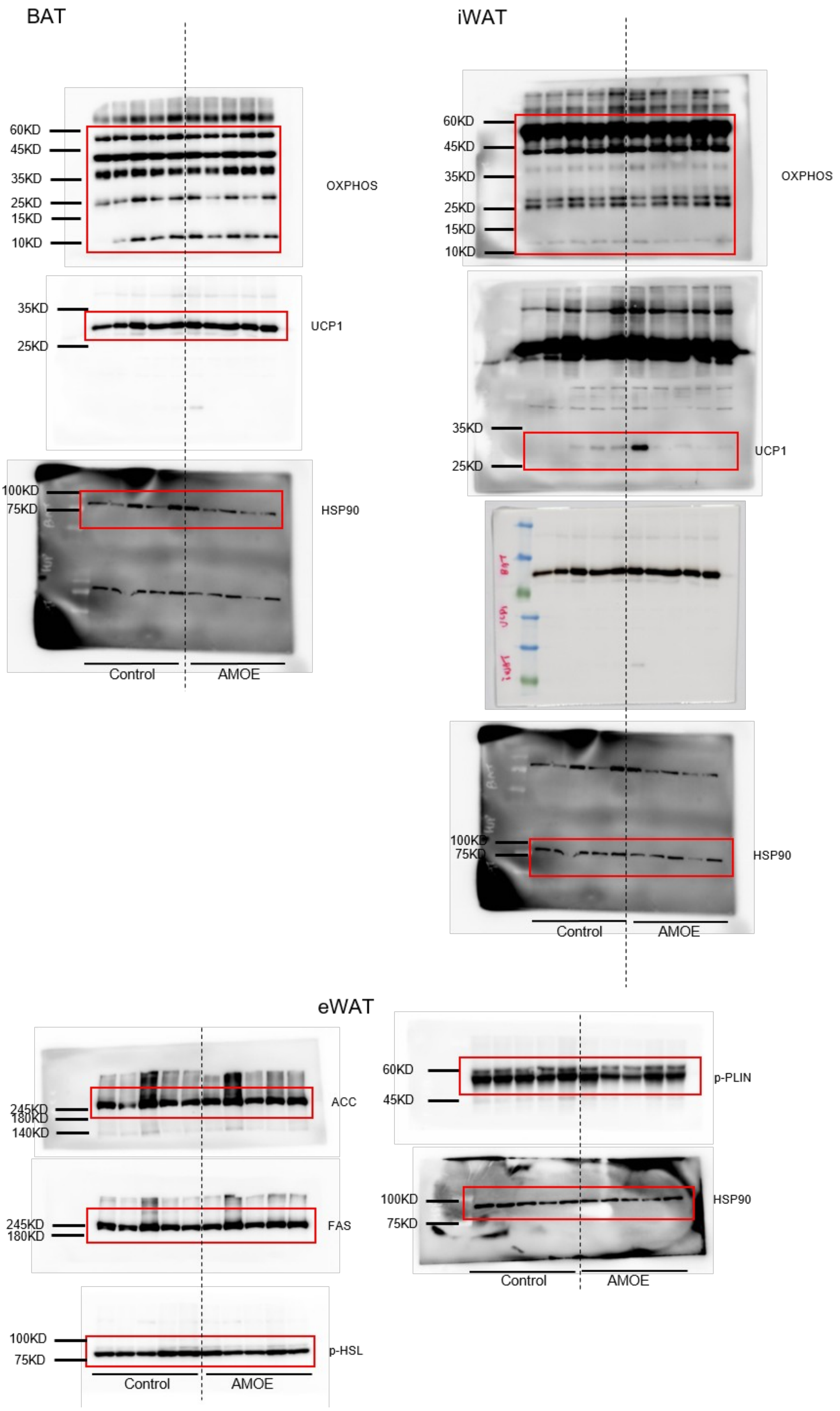
BAT
60KD
45KD
35KD
25KD
15KD
10KD
OXPHOS
35KD
25KD
UCP1
100KD
75KD
HSP90
Control
AMOE
iWAT
60KD
45KD
35KD
25KD
15KD
10KD
OXPHOS
35KD
25KD
UCP1
100KD
75KD
HSP90
Control
AMOE
eWAT
245KD
180KD
140KD
ACC
245KD
180KD
FAS
100KD
75KD
p-HSL
Control
AMOE
60KD
45KD
p-PLIN
100KD
75KD
HSP90
Control
AMOE

Supplementary Figure 2D

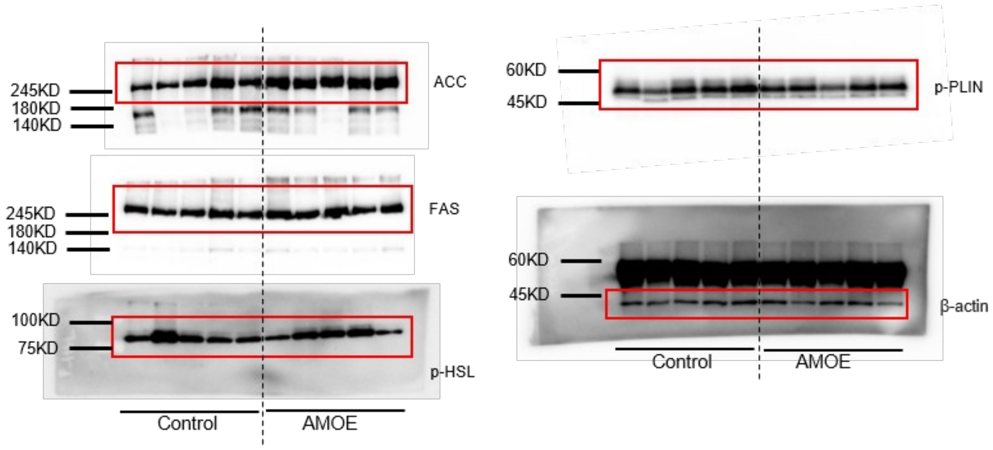

Supplement: Supplementary file 1 [file 42826_2026_296_MOESM2_ESM.docx]
