# Supplementary material for: Adipocyte-specific MC2R overexpression exerts minimal metabolic effects in mice
Source: Lab Anim Res. 2026 Sep 4;42:34. doi: 10.1186/s42826-026-00296-4 (PMC13543462; doi:10.1186/s42826-026-00296-4)
Supplement: Supplementary file 1 [file 42826_2026_296_MOESM1_ESM.docx]

**Supplementary Materials**

**Supplementary Table 1. The qPCR primer sequences used in this study.**

| **Gene** | **Primer** | **5’– 3’ sequence** |
| --- | --- | --- |
| ***Tbp*** | Forward | CCCCTTGTACCCTTCACCAAT |
|  | Reverse | GAAGCTGCGGTACAATTCCAG |
| ***Acaca*** | Forward | ATGGGCGGAATGGTCTCTTTC |
|  | Reverse | TGGGGACCTTGTCTTCATCAT |
| ***Fasn*** | Forward | GGAGGTGGTGATAGCCGGTAT |
|  | Reverse | TGGGTAATCCATAGAGCCCAG |
| ***Lipe*** | Forward | GCTCATCTCCTATGACCTACGG |
|  | Reverse | TCCGTGGATGTGAACAACCAGG |
| ***Pnpla2*** | Forward | GGATGGCGGCATTTCAGACA |
|  | Reverse | CAAAGGGTTGGGTTGGTTCAG |
| ***Ucp1*** | Forward | CAAAAACAGAAGGATTGCCGAAA |
|  | Reverse | TCTTGGACTGAGTCGTAGAGG |
| ***Dio2*** | Forward | AATTATGCCTCGGAGAAGACCG |
|  | Reverse | GGCAGTTGCCTAGTGAAAGGT |
| ***Fgf21*** | Forward | GTGTCAAAGCCTCTAGGTTTCTT |
|  | Reverse | GGTACACATTGTAACCGTCCTC |
| ***Ppargc1a*** | Forward | AGCCGTGACCACTGACAACGAG |
|  | Reverse | GCTGCATGGTTCTGAGTGCTAAG |
| ***Mc2r*** | Forward | ACACCGCAAGAAATAACTCCG |
|  | Reverse | AGGAGGACAATCAAGTTCTCCA |

**Supplementary Figure S1.** **Single-cell analysis of *Mrap* expression in mouse WAT.**

UMAP visualization of *Mrap* expression in mouse WAT using publicly available single-cell RNA-seq data from “A single cell atlas of human and mouse white adipose tissue” (Single Cell Portal dataset SCP1376; GSE176171).

**Supplementary Figure S2. Adipocyte-specific MC2R overexpression in female mice does not induce metabolic changes following exogenous ACTH administration.**

A) Body weight measurements of AMOE female mice after injecting exogenous ACTH (control, n = 7; AMOE, n = 11). B) Adipose tissue weights of AMOE female mice injected with exogenous ACTH (control, n = 7; AMOE, n = 11). C) mRNA expression levels of the indicated genes in the adipose tissues of AMOE female mice after exogenous ACTH administration (control, n = 7; AMOE, n = 7). D) Immunoblot analysis of the indicated proteins in the eWAT of AMOE female mice after ACTH injection (control, n = 5; AMOE, n = 5). β-actin was used as a loading control. A student’s t-test was used to conduct statistical analysis, with values presented as the mean ± SEM. Differences among groups are denoted as * p < 0.05, N.S. = not significant.
